# A Comparison of Conventional Rotating Method and Non-Rotating Method for Double-Lumen Tube Insertion Using a Customized Rigid J-Shaped Stylet for One-Lung Ventilation: A Randomized Controlled Trial

**DOI:** 10.3390/jcm13175302

**Published:** 2024-09-06

**Authors:** Soomin Lee, Sung Joon Han, Jiho Park, Yoon-Hee Kim, Boohwi Hong, Chahyun Oh, Seok-Hwa Yoon

**Affiliations:** 1 Department of Anesthesiology and Pain Medicine, Chungnam National University Hospital, Daejeon 35015, Republic of Korea; bimily0526@gmail.com (S.L.); yhkim040404@gmail.com (Y.-H.K.); koho0127@gmail.com (B.H.); 2 Department of Anesthesiology and Pain Medicine, College of Medicine, Chungnam National University, Daejeon 35015, Republic of Korea; jihopark@cnuh.co.kr; 3 Department of Thoracic and Cardiovascular Surgery, College of Medicine, Chungnam National University Hospital, Daejeon 35015, Republic of Korea; hansungjoon@cnuh.co.kr; 4 Department of Anesthesiology and Pain Medicine, Sejong Chungnam National University Hospital, Sejong 30099, Republic of Korea

**Keywords:** double-lumen tube, video laryngoscopy, non-rotatory technique, thoracic surgery, one-lung ventilation, airway management, intubation success rate, intubation time

## Abstract

**Background**: The conventional double-lumen tube (DLT) insertion method requires a rotatory maneuver that was developed using direct laryngoscopy and may not be optimal for video laryngoscopy. This study compared a new non-rotatory maneuver with the conventional method for DLT insertion using video laryngoscopy. **Methods**: Patients scheduled for thoracic surgery requiring one-lung ventilation were randomly assigned to either the rotating (R) or non-rotating (NR) method groups. All patients were intubated using a customized rigid J-shaped stylet, a video laryngoscope, and a left-sided silicone DLT. The conventional rotatory maneuver was performed in the R group. In the NR group, the stylet was inserted with its tip oriented anteriorly (12 o’clock direction) while maintaining the bronchial lumen towards the left (9 o’clock direction). After reaching the glottic opening, the tube was inserted using a non-rotatory maneuver, maintaining the initial orientation. The primary endpoint was the intubation time. Secondary endpoints included first-trial success rate, sore throat, hoarseness, and airway injury. **Results**: Ninety patients (forty-five in each group) were included. The intubation time was significantly shorter in the NR group compared to the R group (22.0 [17.0, 30.0] s vs. 28.0 [22.0, 34.0] s, respectively), with a median difference of 6 s (95% confidence interval [CI], 3–11 s; *p* = 0.017). The NR group had a higher first-attempt success rate and a lower incidence of sore throats. **Conclusions**: The non-rotatory technique with video laryngoscopy significantly reduced intubation time and improved first-attempt success rate, offering a viable and potentially superior alternative to the conventional rotatory technique.

## 1. Introduction

Double-lumen tubes (DLTs) have become a crucial component in thoracic surgery, especially as more procedures are transitioning to minimally invasive techniques using videoscope-assisted techniques that require one-lung ventilation [1]. While airway management is a fundamental skill for anesthesiologists, DLT intubation requires additional skills and experience due to the tube’s larger size and unique design, which is tailored for lung separation [2].

Traditionally, for left-sided DLT intubation, a rotatory maneuver is used to guide the bronchial tube to the left after passing the glottic opening [3]. However, this technique was primarily developed during the era of direct laryngoscopy, raising questions about its suitability for video laryngoscopy, which has largely replaced direct laryngoscopy. Video laryngoscopy does not provide a direct line of sight or a straight path to the airway; thus, intubation techniques may be adjusted accordingly.

Notably, typical stylets designed for video laryngoscopes have a more acute curvature than those designed for direct laryngoscopes, creating additional challenges for rotatory maneuvers. To address this potential mismatch, we introduced a non-rotatory (NR) technique for DLT insertion. We hypothesized that the non-rotatory maneuver would be more feasible than the rotatory maneuver for DLT insertion using a video laryngoscope.

## 2. Materials and Methods

### 2.1. Study Design and Participants

This single-center, single-blinded (patient), parallel-arm, randomized trial was conducted at Chungnam National University Hospital, a tertiary teaching hospital in the Republic of Korea. This study was approved by the Institutional Review Board of Chungnam National University Hospital (Daejeon, Republic of Korea, IRB number: 2020-02-093, investigator: Seok-Hwa Yoon, approval date: 5 June 2020) and registered at cris.nih.go.kr (KCT0005188, Principal investigator: Soomin Lee, Date of registration: 1 July 2020) before patient enrollment. Written informed consent was obtained from all participants before surgery. 

Eligibility criteria included patients aged 20–70 years, with an American Society of Anesthesiologists (ASA) physical status 1–3, and scheduled for thoracic surgery requiring one-lung ventilation. Exclusion criteria were restricted neck extension, need for rapid sequence intubation, presence of loose or vulnerable teeth, tracheobronchial abnormalities, body mass index (BMI) ≥35 kg/m², interincisor distance <3 cm, thyromental distance <6 cm, and Mallampati grade 4.

Study data were collected and managed using Research Electronic Data Capture (REDCap) software (Version 6.11.5) hosted at Chungnam National University Hospital. REDCap is a secure web-based platform designed to capture data for research studies. This study adheres to the applicable CONSORT (Consolidated Standards for Reporting Trials) guidelines.

### 2.2. Randomization and Blinding

Patients were randomly assigned at a ratio of 1:1 to either the rotatory (R; conventional method) or non-rotatory (NR; new method) groups. Block randomization with sizes of three and six was performed. To ensure allocation concealment, the sequence was uploaded to REDCap (http://redcap.cnuh.co.kr) and revealed to the physician performing DLT insertion immediately before each procedure. Except for the researcher recording the intubation process, all other researchers involved in outcome assessments and the participants were blinded to the group assignments.

### 2.3. Intervention

**Figure 1:**
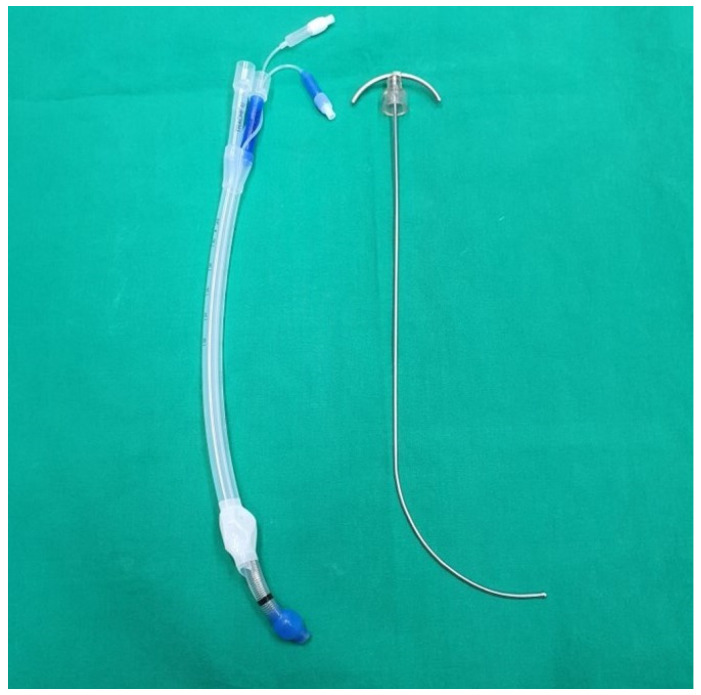
Left-sided silicone double-lumen endotracheal tube (Human Broncho, INSUNG Medical Co., Ltd., Wonju, Republic of Korea) and a customized rigid J-shaped stylet. The stylet was designed with the same curvature as the UESCOPE^®^ stylet for a single-lumen tube and was long enough to reach the end of the bronchial tip.

All patients were intubated using a video laryngoscope (UESCOPE^®^ VL310, Zhejiang UE Medical Corp., Zhejiang, China) and a left-sided silicone double-lumen endotracheal tube (Human Broncho, INSUNG Medical Co., Ltd., Wonju, Republic of Korea) with a customized rigid J-shaped stylet (Figure 1). The stylet was designed with the same curvature as the UESCOPE^®^ stylet for a single-lumen tube and was long enough to reach the end of the bronchial tip. The view of the glottic opening via video laryngoscopy was scored using the Cormack-Lehane grade. All intubations were performed by senior residents proficient in video laryngoscope-assisted single-lumen tube insertion and with at least 20 experiences with DLT insertion using the conventional method. Prior to the study, these residents were provided with instructions and simulation training sessions to familiarize themselves with the NR technique.

**Figure 2:**
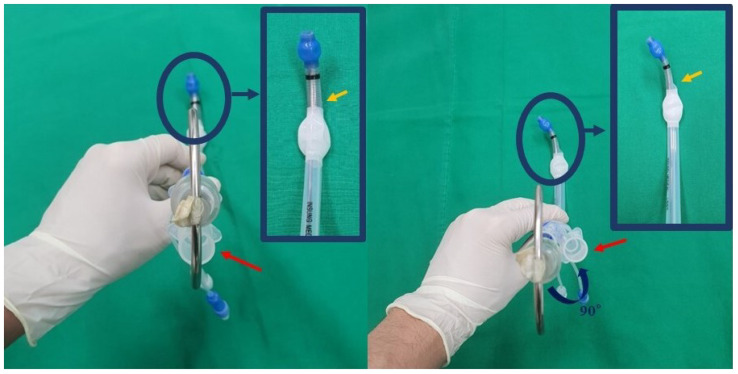
The conventional method (rotatory maneuver) for double-lumen tube insertion. Initially, the stylet and bronchial lumen were aligned and oriented anteriorly (left-side image). The proximal (red arrow) and distal (yellow arrow) orifices of the tracheal lumen were oriented posteriorly. As the bronchial lumen of the tube passed through the vocal cords, the tube was rotated 90° counterclockwise (right-side image), with the proximal and distal orifices of the tracheal lumen now oriented to the right side.

In the R group, the stylet was inserted with the J-shaped tip aligned with the left-sided bronchial opening of the tube, and the tip was kept facing anteriorly in the 12 o’clock direction. Subsequently, as the bronchial lumen of the tube passed through the vocal cords, the tube was rotated 90° counterclockwise (Figure 2, Video S1).

**Figure 3:**
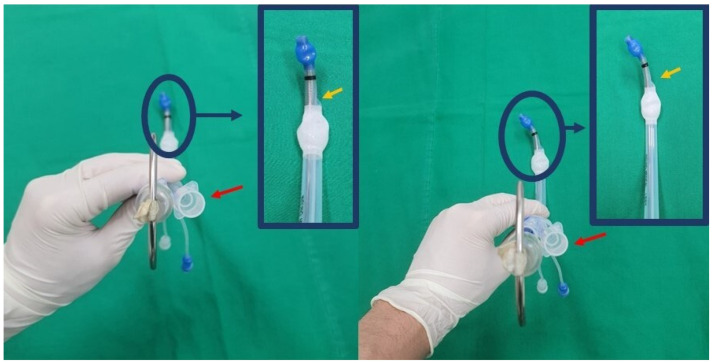
The new method (non-rotatory maneuver) for double-lumen tube insertion. Initially, the stylet was inserted with its tip oriented anteriorly while maintaining the bronchial lumen of the tube oriented towards the left (left-side image). The proximal (red arrow) and distal (yellow arrow) orifices of the tracheal lumen were oriented to the right side. The tube was inserted using a non-rotatory maneuver (right-side image), keeping the proximal and distal orifices oriented to the right side.

In the NR group, the stylet was inserted with its tip oriented anteriorly (12 o’clock direction) while maintaining the bronchial lumen of the tube oriented towards the left (9 o’clock direction). The tube was inserted using a non-rotatory maneuver (Figure 3, Video S2).

### 2.4. Anesthetic Procedures

Patients received premedication with intramuscular injections of glycopyrrolate (0.2 mg/kg) and midazolam (2 mg) 30 min before surgery. General anesthesia was administered with standard ASA monitoring. After a 3 min pre-oxygenation, general anesthesia was induced using propofol (1–2 mg/kg), remifentanil (1 µg/kg), and rocuronium (0.6 mg/kg). Endotracheal intubation was then performed using the allocated maneuver. The tube cuff pressure was set to 20–30 mmHg, with the tidal volume adjusted to 6–8 mL/kg of the ideal body weight and target end-tidal CO2 set to 35–45 mmHg. Anesthesia was maintained with sevoflurane inhalation and a continuous infusion of remifentanil (0.05–0.2 μg/kg/min).

### 2.5. Outcome Measures

The primary endpoint was the intubation time (T_total_), defined as the sum of the time taken from the insertion of the video laryngoscope until visualization of the vocal cord (T_1_) and the time from the tube passing the lips until the end of tube insertion (T_2_). Secondary endpoints included the success rate at the first intubation trial, sore throat, hoarseness, and airway injuries. 

Postoperative outcomes, including sore throat, hoarseness, and airway injury, were evaluated by a researcher blinded to the group assignments. Sore throat and hoarseness were assessed 30 min and 24 h postoperatively using a four-point scale (none, mild, moderate, and severe). Airway injury was evaluated postoperatively while the patient was anesthetized and intubated in the supine position. The DLT was withdrawn until the bronchial lumen was within the trachea. Then, a fiberoptic bronchoscope was used to evaluate both sides of the bronchus, carina, and distal tracheal lumen. Any findings, such as redness, edema, and hematoma, were recorded.

### 2.6. Statistical Analysis

All statistical analyses were performed on an intention-to-treat basis using R software version 4.0.3 (R Project for Statistical Computing, Vienna, Austria). The sample size was calculated using G*Power software (version 3.2.1; Kiel, Germany). We referred to the effect size of 0.74 from a previous study [4], which compared the intubation time of two different techniques. To achieve a power of 0.9 and an alpha of 0.05, 40 participants per group were needed. Considering a dropout rate of 10%, 90 patients were finally recruited. 

Continuous variables were analyzed using either the independent *t*-test (mean ± SD) or the Mann–Whitney U test (median [1Q, 3Q]), based on the Shapiro–Wilk test for normality of data distribution. For the primary endpoint, estimation statistics were used to assess the effect size, employing bootstrap resampling techniques to calculate 95% confidence intervals (CIs) and mean or median differences between groups [5]. Categorical variables were analyzed using the χ² test or Fisher’s exact test (for expected counts <5) and reported as numbers (percentages). A two-tailed *p*-value < 0.05 was considered statistically significant.

## 3. Results

**Figure 4:**
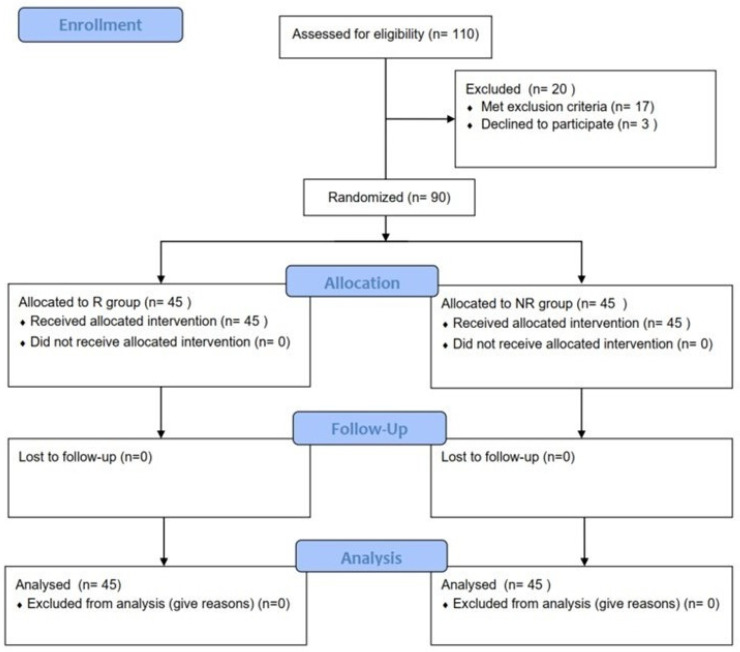
CONSORT flow diagram. R group, rotatory (conventional method) group; NR group, non-rotatory (new method) group.

From July 2020 to August 2021, a total of 110 patients were assessed for eligibility, with 90 enrolled in the study and randomly assigned. All enrolled patients were included in the final analysis (Figure 4). Table 1 summarizes the baseline characteristics of the patients included in the final analysis.

**Figure 5:**
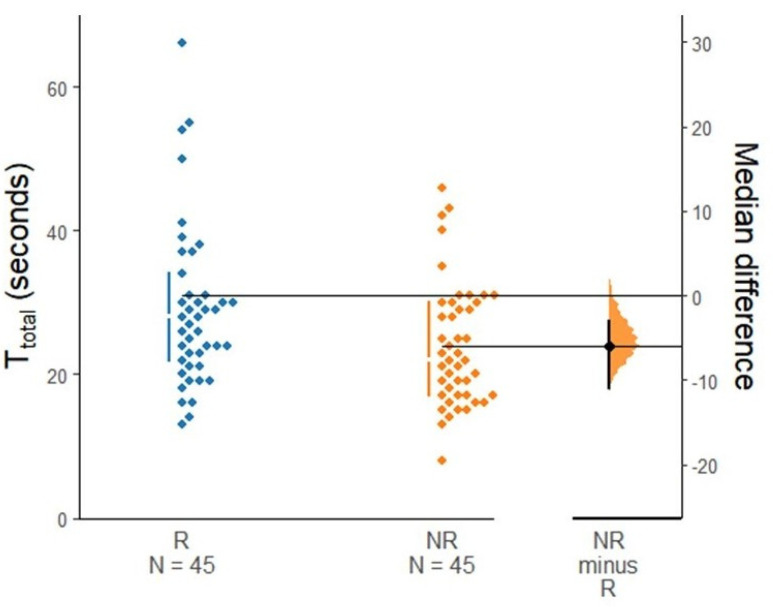
The intubation time (T_total_) using conventional (R, rotatory) and the new (NR, non-rotatory) maneuvers. Left panel: Each dot represents the T_total_ values of individual patients stratified by group, with the gapped lines on the left indicating the median (gap) and interquartile range (vertical end). Right panel: Effect size is plotted with the median difference (dot) and 95% confidence intervals (ends of the vertical error bar).

The T_total_ was significantly shorter in the NR group than in the R group (22.0 [17.0, 30.0] s vs. 28.0 [22.0, 34.0] s, respectively) with a median difference of 6 s (95% CI, 3–11 s; *p* = 0.017, Figure 5). Specifically, T_2_ was significantly shorter in the NR group (14.0 [12.0, 20.0] s vs. 19.0 [17.0, 25.0] s, *p* < 0.001). Additionally, the success rate at the first trial of intubation was also higher in the NR group (95.6% vs. 77.8%, *p* = 0.030). The incidence of sore throat was significantly lower in the NR group. Other secondary outcomes showed no significant difference. Table 2 summarizes the outcomes.

## 4. Discussion

This study aimed to evaluate a novel non-rotatory technique for DLT insertion using a video laryngoscope and compare it with the conventional rotatory method. Our findings demonstrated that the NR method significantly reduced intubation time and improved the first-attempt success rate without increasing complications, suggesting that this new technique is a viable alternative in clinical practice.

Recently, video laryngoscopy has become widely used, providing easier intubation of single-lumen tubes, reducing failed intubation rates, and increasing first-attempt success due to better glottic visualization, offering a safer risk profile than direct laryngoscopy for adults [6,7]. However, the use of video laryngoscopy for DLT insertion remains controversial [8]. A 2018 meta-analysis showed that, while video laryngoscopy provided higher first-attempt success rates with a lower incidence of oral, mucosal, or dental injuries compared to direct laryngoscopy, it did not demonstrate any advantage in terms of intubation time. Additionally, video laryngoscopy resulted in a higher incidence of malpositioned DLT. This may suggest that the rotatory maneuver is suboptimal for video laryngoscope-assisted intubation.

Our study introduced a non-rotatory technique potentially better suited for video laryngoscopy. This technique eliminates the cumbersome 90° rotation required by the conventional method, which can be challenging given the indirect visualization and limited working space afforded by video laryngoscopy. The simplified NR approach facilitated more efficient and accurate DLT placement, as evidenced by the reduced intubation time and higher first-attempt success rates observed in our study. Moreover, the high success rate achieved by senior residents with no prior experience in the new method, apart from brief instruction and simulation sessions, underscores the practicality and ease of adoption of the NR technique.

DLTs, being more rigid and larger than traditional single-lumen tubes, present additional challenges during intubation, increasing the likelihood of complications related to insertion and secure positioning [9,10,11,12]. The NR technique eliminates the need for this rotation, simplifying the insertion process and potentially reducing the apnea duration in patients. Additionally, familiarity with single-lumen endotracheal tube intubation makes the NR technique easier to perform, thus facilitating a smoother learning curve.

However, if this new method increases the incidence of adverse effects such as airway trauma, hoarseness, and postoperative sore throat, it would not be a viable alternative. Our results showed a lower incidence of sore throat with the new method and no significant difference in the rate of airway injury. In this study, we used a DLT with a flexible wired bronchial tip, which likely minimized the risk of airway injury. The lower incidence of sore throats may be due to the avoidance of tube rotation near the vocal cords, thus reducing friction and irritation. Even without a rotatory maneuver, the wired bronchial lumen tip, naturally curved to the left, smoothly entered the left bronchus, showing a higher rate of first-attempt success.

Patients undergoing thoracic surgery often have poor lung function and limited tolerance to apnea [13]. Therefore, the rapid and precise positioning of the DLT is crucial for these patients. Recently, numerous studies have addressed this issue. Some studies compared different angulations of the shaft or tip of the DLT using direct laryngoscopy [4,14], while others compared different types of video laryngoscopes for DLT insertion [15,16], or evaluated direct versus video laryngoscopy [17,18,19]. However, no studies have explored DLT insertion without using a rotation technique. As the recent and upcoming generations of anesthesiologists have become more familiar with video laryngoscopy than with direct laryngoscopy, various approaches using video laryngoscopy, such as those in this study, will be increasingly important.

Although our study provides promising results, it has notable limitations. First, its applicability in patients with difficult airways or obesity remains uncertain. Second, we used a specific type of DLT with a wired bronchial tip, video laryngoscope, and customized stylet; therefore, our findings may not apply to other types of tubes or direct laryngoscopes. Additionally, the stylet used with a video laryngoscope should have a curvature that aligns properly with the curvature of the scope. The generalizability of our findings to other video laryngoscope models has not yet been established. Finally, the study was conducted at a single center with a limited sample size, which may have affected the generalizability of the findings. Caution is advised, and further research is necessary to address these limitations.

## 5. Conclusions

The non-rotatory maneuver for DLT insertion using a video laryngoscope offers a viable and potentially superior alternative to the conventional rotatory technique. By simplifying the insertion process, improving success rates, ensuring proper placement, and maintaining a comparable safety profile, this new method holds promise for enhancing the clinical practice in thoracic anesthesia.

## Figures and Tables

**Table 1:** Baseline characteristics.

	R Group	NR Group
	(*n* = 45)	(*n* = 45)
Age (yr)	62.0 (44.0, 66.0)	59.0 (47.0, 65.0)
Sex (female)	14 (31.1)	16 (35.6)
Height (cm)	165.6 ± 9.7	164.9 ± 9.2
Weight (kg)	62.1 (56.2, 68.7)	63.0 (56.1, 69.7)
BMI (kg/m^2^)	23.3 ± 3.9	23.4 ± 3.4
ASA (>2)	10 (22.2)	7 (15.6)
Surgery type		
Lung resection	36 (80.0)	33 (73.3)
Mediastinal procedure	7 (15.6)	9 (20.0)
Others	2 (4.4)	3 (6.7)
Procedure side		
Bilateral	1 (2.2)	1 (2.2)
Left	29 (64.4)	19 (42.2)
Right	15 (33.3)	25 (55.6)
Mallampati grade		
1	27 (60.0)	20 (44.4)
2	12 (26.7)	18 (40.0)
3	6 (13.3)	7 (15.6)
Cormack-Lehane grade		
1	29 (65.9)	24 (53.3)
2	11 (25.0)	17 (37.8)
3	4 (9.1)	3 (6.7)
4	0 (0.0)	1 (2.2)
Interincisor distance (cm)	4.1 (4.0, 5.0)	4.0 (4.0, 4.5)
Thyromental distance (cm)	8.0 (7.0, 9.0)	8.0 (7.4, 9.0)
Tube size (Fr)		
35	3 (6.7)	2 (4.4)
37	20 (44.4)	25 (55.6)
39	22 (48.9)	18 (40.0)
Anesthesia duration (min)	110.0 (71.0, 176.0)	100.0 (68.0, 174.0)

Values are mean ± standard deviation, median (1Q, 3Q), or number (%). No notable difference in the clinical characteristics was found between groups. Abbreviations: R group, rotatory (conventional method) group; NR group, non-rotatory (new method) group; BMI, body mass index; ASA, American Society of Anesthesiologists physical status.

**Table 2:** Summary of the outcomes.

	R Group	NR Group	*p*
	(*n* = 45)	(*n* = 45)	
Primary outcome			
T_total_ (seconds)	28.0 (22.0, 34.0)	22.0 (17.0, 30.0)	0.017
T_1_ (seconds)	7.0 (5.0, 10.0)	7.0 (4.0, 9.0)	0.629
T_2_ (seconds)	19.0 (17.0, 25.0)	14.0 (12.0, 20.0)	<0.001
Secondary outcomes			
Success at 1st trial	35 (77.8)	43 (95.6)	0.030
Sore throat at 30 min			0.043
None	18 (40.0)	29 (64.4)	
Mild	26 (57.8)	15 (33.3)	
Moderate	1 (2.2)	1 (2.2)	
Sore throat at 24 h			0.087
None	34 (75.6)	40 (88.9)	
Mild	11 (24.4)	4 (8.9)	
Moderate	0 (0.0)	1 (2.2)	
Sore throat, overall	27 (60.0)	16 (35.6)	0.035
Hoarseness at 30 min			0.211
None	20 (44.4)	29 (64.4)	
Mild	22 (48.9)	14 (31.1)	
Moderate	2 (4.4)	2 (4.4)	
Severe	1 (2.2)	0 (0.0)	
Hoarseness at 24 h			1.000
None	40 (88.9)	39 (86.7)	
Mild	5 (11.1)	6 (13.3)	
Hoarseness, overall	25 (55.6)	17 (37.8)	0.139
Airway injury			0.586
None	26 (57.8)	29 (64.4)	
Edema	0 (0.0)	1 (2.2)	
Redness	18 (40.0)	15 (33.3)	
Hematoma	1 (2.2)	0 (0.0)	

Values are medians (1Q, 3Q) or numbers (%). The primary endpoint was the intubation time (T_total_), defined as the sum of the time taken from the insertion of the video laryngoscope until visualization of the vocal cord (T_1_) and the time from the tube passing the lips until the end of tube insertion (T_2_). Abbreviations: R group, rotatory (conventional method) group; NR group, non-rotatory (new method) group.

## Data Availability

The original contributions presented in the study are included in the article. Further inquiries can be directed to the corresponding authors.

## Supplementary Materials

The following supporting information can be downloaded at: https://www.mdpi.com/article/10.3390/jcm13175302/s1, Video S1: R group: Conventional; Rotatory group; Video S2: NR group: New; Non-Rotatory group.
